# Supplementary material for: Goat’s Skim Milk Enriched with Agrocybe aegerita (V. Brig.) Vizzini Mushroom Extract: Optimization, Physico-Chemical Characterization, and Evaluation of Techno-Functional, Biological and Antimicrobial Properties
Source: Foods. 2025 Mar 19;14(6):1056. doi: 10.3390/foods14061056 (PMC11942284; doi:10.3390/foods14061056)
Supplement: Supplementary file 1 [file foods-14-01056-s001.zip › foods-3502354-supplementary.pdf]

Article

Goat's Skim Milk Enriched with *Agrocybe aegerita* (V. Brig.) Vizzini Mushroom Extract: Optimization, Physico-Chemical Characterization, and Evaluation of Techno-Functional, Biological and Antimicrobial Properties

Danijel D. Milinčić [1,†], Ivana Sredović Ignjatović [1,†], Dejan Stojković [2], Jovana Petrović [2], Aleksandar Ž. Kostić [1], Jasmina Glamočlija [2], Ana Doroški Petković [1], Ana Plećić [1], Steva Lević [1], Vladislav Rac [1], Vladimir B. Pavlović [1], Slađana P. Stanojević [1], Viktor A. Nedović [1] and Mirjana B. Pešić [1,*]

[1] Institute of Food Technology and Biochemistry, Faculty of Agriculture, University of Belgrade, Nemanjina 6, 11080 Belgrade, Serbia; danijel.milincic@agrif.bg.ac.rs (D.D.M.); isredovic@agrif.bg.ac.rs (I.S.I.); akostic@agrif.bg.ac.rs (A.Ž.K.); ana.doroski@agrif.bg.ac.rs (A.D.P.); ana.bjekovic96@gmail.com (A.P.); slevic@agrif.bg.ac.rs (S.L.); vladarac@agrif.bg.ac.rs (V.R.); vlaver@agrif.bg.ac.rs (V.B.P.); sladjas@agrif.bg.ac.rs (S.P.S.); vnedovic@agrif.bg.ac.rs (V.A.N.)

[2] Institute for Biological Research, "Siniša Stanković"—National Institute of the Republic of Serbia,
University of Belgrade, Bulevar Despota Stefana 142, 11108 Belgrade, Serbia; dejanbio@ibiss.bg.ac.rs (D.S.); jovana0303@ibiss.bg.ac.rs (J.P.); jasna@ibiss.bg.ac.rs (J.G.)

* Correspondence: mpesic@agrif.bg.ac.rs; Tel.: +381-114413315

† These authors contributed equally to this work.

Table S1. Estimated regression coefficients and analysis of variance for total **phenolic content** (Folin Ciocalteu method-TPC) response design.

Estimated regression coefficients for the response surface design						
Term	Coefficient	Standard error of the coefficient				*p*-value
Constant	48.2507	1.310				0.000
w(ME), %	2.3867	1.288				0.106
pH	2.4117	1.288				0.103
w(ME) * *w*(ME)	-0.7924	1.899				0.689
pH * pH	2.0726	1.899				0.311
w(ME) * pH	1.4225	1.578				0.397
Analysis of variance (ANOVA) for response surface model (coded units)						
Source of variation	Degrees of freedom	Sequential sum of squares	Adjusted sum of squares	Adjusted mean of squares	*F*-value	*p*-value
Regression	5	89.032	89.032	17.806	1.79	0.234
Linear	2	69.074	69.074	34.537	3.47	0.090
Square	2	11.864	11.864	5.932	0.600	0.577
Interaction	1	8.094	8.094	8.094	0.810	0.397
Residual Error	7	69.707	69.707	9.958		
Lack-of-Fit	3	64.452	64.452	21.484	16.350	0.010
Pure Error	4	5.256	5.256	1.314		
Total	12	158.739				

Table S2. Estimated regression coefficients and analysis of variance for **ABTS**$^{•+}$ (ABTS$^{•+}$ radical scavenging activity) response design.

Estimated regression coefficients for the response surface design			
Term	Coefficient	Standard error of the coefficient	*p*-value
Constant	42.4855	1.393	0.000
w(ME), %	0.7333	1.370	0.609
pH	6.1383	1.370	0.003
w(ME) * *w*(ME)	-2.7793	2.019	0.211
pH * pH	2.7757	2.019	0.212
w(ME) * pH	3.1500	1.678	0.103

Analysis of variance (ANOVA) for response surface model (coded units)						
Source of variation	Degrees of freedom	Sequential sum of squares	Adjusted sum of squares	Adjusted mean of squares	*F*-value	*p*-value
Regression	5	299.850	299.850	59.970	5.33	0.024
Linear	2	229.301	229.301	114.651	10.19	0.008
Square	2	30.858	30.858	15.429	1.37	0.315
Interaction	1	39.690	39.690	39.690	3.53	0.103
Residual Error	7	78.797	78.797	11.257		
Lack-of-Fit	3	48.462	48.462	16.154	2.13	0.239
Pure Error	4	30.334	30.334	7.584		
Total	12	378.646				

Table S3. Estimated regression coefficients and analysis of variance for **DPPH**$^{•}$ (DPPH$^{•}$ radical scavenging activity) response design.

Estimated regression coefficients for the response surface design			
Term	Coefficient	Standard error of the coefficient	*p*-value
Constant	27.4121	0.5675	0.000
w(ME), %	6.3550	0.5580	0.000
pH	-0.6300	0.5580	0.296
w(ME) * *w*(ME)	-3.6122	0.8224	0.003
pH * pH	0.2828	0.8224	0.741
w(ME) * pH	-0.3300	0.6834	0.644

Analysis of variance (ANOVA) for response surface model (coded units)						
Source of variation	Degrees of freedom	Sequential sum of squares	Adjusted sum of squares	Adjusted mean of squares	*F*-value	*p*-value
Regression	5	285.033	285.033	57.007	30.52	0.000
Linear	2	244.698	244.698	122.349	65.50	0.000
Square	2	39.900	39.900	19.950	10.68	0.007
Interaction	1	0.436	0.436	0.436	0.23	0.644
Residual Error	7	13.075	13.075	1.868		
Lack-of-Fit	3	7.352	7.352	2.451	1.71	0.302
Pure Error	4	5.724	5.724	1.431		
Total	12	298.109				

Table S4. Estimated regression coefficients and analysis of variance for **ferric reducing power** (FRP) response design.

Estimated regression coefficients for the response surface design						
Term	Coefficient		Standard error of the coefficient			*p*-value
Constant	36.9403		1.688			0.000
w(ME), %	20.8633		1.659			0.000
pH	0.4750		1.659			0.783
w(ME) * *w*(ME)	-6.9062		2.445			0.026
pH * pH	5.6888		2.445			0.053
w(ME) * pH	-0.5875		2.032			0.781
Analysis of variance (ANOVA) for response surface model (coded units)						
Source of variation	Degrees of freedom	Sequential sum of squares	Adjusted sum of squares	Adjusted mean of squares	*F*-value	*p*-value
Regression	5	2776.35	2776.35	555.27	33.62	0.000
Linear	2	2613.03	2613.03	1306.51	79.10	0.000
Square	2	161.94	161.94	4.90	80.97	0.047
Interaction	1	1.38	1.38	1.38	0.08	0.781
Residual Error	7	115.62	115.62	16.52		
Lack-of-Fit	3	57.78	57.78	19.26	1.33	0.382
Pure Error	4	57.84	57.84	14.46		
Total	12	2891.97				

Table S5. Estimated regression coefficients and analysis of variance for **ferrous chelating capacity** (CHE) response design.

Estimated regression coefficients for the response surface design						
Term	Coefficient		Standard error of the coefficient			*p*-value
Constant	141.987		3.785			0.000
w(ME), %	34.570		3.721			0.000
pH	-1.003		3.721			0.795
w(ME) * *w*(ME)	-21.438		5.484			0.006
pH * pH	-25.938		5.484			0.002
w(ME) * pH	-6.080		4.557			0.224
Analysis of variance (ANOVA) for response surface model (coded units)						
Source of variation	Degrees of freedom	Sequential sum of squares	Adjusted sum of squares	Adjusted mean of squares	*F*-value	*p*-value
Regression	5	12351.6	12351.6	2470.31	29.74	0.000
Linear	2	7176.5	7176.5	3588.27	43.20	0.000
Square	2	5027.1	5027.1	2513.57	30.26	0.000
Interaction	1	147.9	147.9	147.87	1.78	0.224
Residual Error	7	581.5	581.5	83.07		
Lack-of-Fit	3	576.4	576.4	192.12	149.49	0.000
Pure Error	4	5.1	5.1	1.29		
Total	12	12933.1				

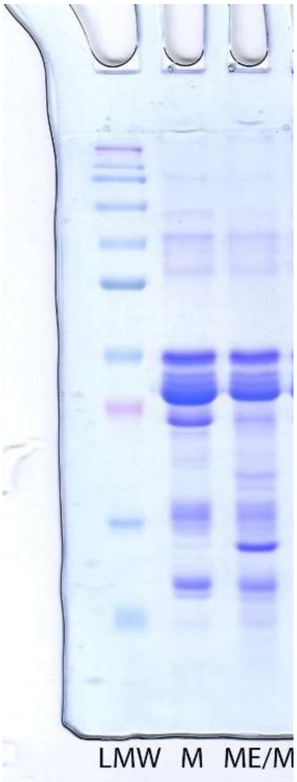

Figure S1. Raw SDS-PAGE gels of the optimized ME/M ingredient and the control M powder. Molecular weight standard (LMW). M - thermally treated skim goat milk; ME/M-optimized mushroom extract/milk mixture.
